# The Influence of Microbiome Dysbiosis and Bacterial Biofilms on Epidermal Barrier Function in Atopic Dermatitis—An Update

**DOI:** 10.3390/ijms22168403

**Published:** 2021-08-05

**Authors:** Leszek Blicharz, Lidia Rudnicka, Joanna Czuwara, Anna Waśkiel-Burnat, Mohamad Goldust, Małgorzata Olszewska, Zbigniew Samochocki

**Affiliations:** 1 Department of Dermatology, Medical University of Warsaw, 02-008 Warsaw, Poland; lidia.rudnicka@wum.edu.pl (L.R.); joanna.czuwara@wum.edu.pl (J.C.); anna.waskiel@wum.edu.pl (A.W.-B.); malgorzata.olszewska@wum.edu.pl (M.O.); zbigniew.samochocki@wum.edu.pl (Z.S.); 2 Department of Dermatology, University Medical Center of the Johannes Gutenberg University, 55131 Mainz, Germany; mgoldust@uni-mainz.de

**Keywords:** atopic dermatitis, biofilms, microbiome, staphylococci, skin barrier

## Abstract

Atopic dermatitis (AD) is a common inflammatory dermatosis affecting up to 30% of children and 10% of adults worldwide. AD is primarily driven by an epidermal barrier defect which triggers immune dysregulation within the skin. According to recent research such phenomena are closely related to the microbial dysbiosis of the skin. There is growing evidence that cutaneous microbiota and bacterial biofilms negatively affect skin barrier function, contributing to the onset and exacerbation of AD. This review summarizes the latest data on the mechanisms leading to microbiome dysbiosis and biofilm formation in AD, and the influence of these phenomena on skin barrier function.

## 1. Introduction

Atopic dermatitis (AD) is a chronic inflammatory skin disorder characterized by a highly heterogenous clinical picture [1,2]. No specific tests ensure the diagnosis of AD. Therefore, it is based on clinical criteria (i.e., UK Working Party criteria and the Hanifin and Rajka criteria applicable to the pediatric and adult populations, respectively) [3,4]. The essential features of AD include the presence of eczematous lesions, itch, and a chronic or relapsing disease course [2,5]. The distribution and morphology of skin lesions reveals age-dependent variations. Children under 2 years of age typically present with poorly defined, exudative erythematous lesions located on the trunk, face, and cheeks. The majority of pediatric patients >2 years of age exhibit a more localized eczema affecting flexor surfaces and the lichenification of chronic lesions. As regards adults, AD most commonly manifests as chronic hand eczema, flexural dermatitis, and/or head and neck dermatitis. A recently published paper describing the prevalence of AD in adults revealed that the classic adult type, with lichenified/exudative flexural dermatitis frequently associated with head and neck eczema or hand eczema was the most common AD phenotype [6]. Furthermore, the predominant manifestations of adult-onset AD included nummular eczema-like and prurigo-like phenotypes, whereas childhood-onset AD was more often characterized by lichenified/exudative flexural dermatitis alone and/or in association with portrait dermatitis.

The lack of causative treatment, significant psychosocial impact, and high prevalence reaching 10% of adults and 30% of children make AD a considerable challenge for public health worldwide [7,8].

The current view on AD pathogenesis encompasses the following factors [2,9,10]:

1. An epidermal barrier defect;
2. Immune dysregulation;
3. Skin microbial dysbiosis;
4. The itch/scratch cycle.

**Figure 1:**
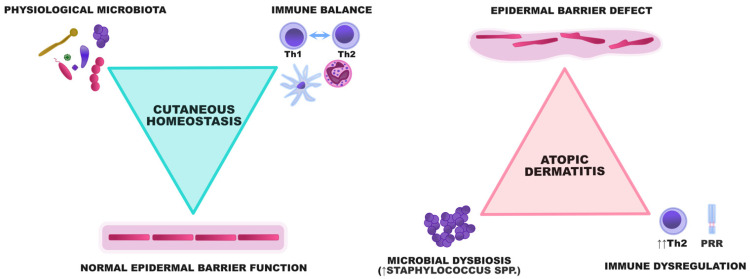
A graphical representation of the interactions between the epidermal barrier, microbiota, and the immune system. In healthy individuals, the epidermis constitutes a barrier preventing the uncontrolled proliferation of potentially harmful microorganisms. A diverse array of microbes participates in the crosstalk with the immune system and ensures its maturation to maintain immune balance within the skin. In AD, the epidermal barrier defect and dysregulation of innate and acquired immunity result in the enhanced adhesion and proliferation of some bacterial species, especially staphylococci. Microbial dysbiosis subsequently aggravates the skin defect by degrading the components of the epidermal barrier and potentiating Th2-skewing.

These pathogenic events are strictly related and trigger the occurrence and exacerbations of AD, making it a model disease for the study of interactions between the skin barrier and cutaneous microbiome (Figure 1).

Literature data suggest that an epidermal barrier defect is the primary factor driving AD symptoms [11,12]. Individuals with AD exhibit highly variable congenital abnormalities that translate into epidermal barrier impairment. The most frequent polymorphisms affect the expression of filaggrin, tight junctions, epidermal lipids, and endothelial protease activity [13,14,15,16,17,18]. The epidermal barrier may also be secondarily damaged by epigenetic changes and environmental factors [2,13,19].

Recent research in the field of the microbiome has highlighted the crucial role of skin microbes in preserving the intact skin barrier [20,21]. Commensal microorganisms are involved in constant crosstalk with the keratinocytes and the immune system, mediate the formation of tight junctions, maintain immune homeostasis, and “teach” the host to recognize and combat pathogens capable of causing a potential skin infection. Furthermore, the physiological microbiome constitutes an additional physiological barrier against pathogenic microbes by competing for the niches favoring their growth [22].

Microbes may function in a sessile form, but in most cases they form complex multi-species communities within the biofilms [23]. A growing body of evidence points to bacterial biofilms as the primary pathogenic factor in a number of dermatological conditions, e.g., in acne vulgaris [24] and chronic wounds [25]. The importance of bacterial biofilms for the skin barrier in AD has also been recently suggested.

The aim of this narrative review is to summarize the recent insights into the interactions between cutaneous microbes, their biofilms, and skin barrier function in AD.

## 2. Materials and Methods

The literature search was performed with the use of the following databases: PubMed, Scopus and Web of Science. The keywords used to perform the search were “atopic dermatitis”, “biofilm”, “microbiome”, “*S. aureus*”, “epidermal differentiation complex”, “tight junctions”, “epidermal proteases”, and “epidermal lipids” in different combinations. There were no language limitations.

## 3. The Function and Structure of the Physiological Epidermal Barrier

The epidermis is a specialized structure that warrants selective permeability for a wide range of exogenous substances and protects the host from dehydration, infection, loss of endogenous molecules, and negative influence of physical factors (e.g., UV radiation, temperature changes, and mechanical stress) [26,27]. The epidermis is formed by keratinocytes which constantly divide in the basal layer and undergo a gradual differentiation while migrating through the more superficial stratum spinosum and stratum granulosum, and eventually slough once they reach the stratum corneum [27]. The physiological turnover time, i.e., the period during which the keratinocyte reaches the stratum corneum, is approximately 28 days. Epidermal differentiation and desquamation are complex processes that may be altered in various dermatoses, e.g., in AD and psoriasis [28].

The integrity of the epidermal barrier is largely ensured by the stratum granulosum and stratum corneum [18,29,30]. The expression of tight junctions (TJs) is one of the most important functional features of the stratum granulosum [26]. Keratinocytes in this layer are also characterized by the presence of keratohyalin granules containing filaggrin and loricrin precursors which, once modified, ensure the appropriate structure of keratinocytes in the stratum corneum.

The keratinocytes in the stratum corneum are referred to as corneocytes. Corneocytes are flat anucleate cells surrounded by a cornified envelope, a structure composed of highly crosslinked insoluble proteins that provide adequate cell structure and adhesion [29]. Intracellular space is filled with lipid lamellae that prevent transepidermal water loss (TEWL) and ensure adequate skin hydration [31].

The crucial elements providing the integrity of the epidermal barrier which might be affected by microbial dysbiosis will be briefly discussed below.

### 3.1. Tight Junctions

The physiological barrier function of the epidermis is largely due to the presence of tight junctions (TJs) that connect the epidermal cells within the stratum granulosum. Their complex structure involves strands between the adjacent keratinocytes formed by claudins, transmembrane proteins primarily composed of occludin (Ocln), JAM-A, and angulin, and proteins forming a “scaffold” within the intracellular zone (zonula occludens (ZO)-1, -2 and -3) [26,32]. Initially, TJs were solely regarded as structures regulating the paracellular diffusion of substances based on their electric charge and molecular weight. Therefore, TJs used to be perceived mainly as a barrier controlling the penetration of potentially dangerous exogenous molecules into the dermis. However, recent advances suggest the key importance of TJs in other aspects of skin physiology, such as the maintenance of the basoapical polarity of the epidermis and the influence on gene expression in the epidermal cells [32,33]. TJs are associated with several cellular signaling networks that regulate cell proliferation and junction assembly. Importantly, different components of the tight junctions are targeted by a wide range of pathogens, which degrade them to cross tissue barriers [34].

Polymorphisms in genes encoding TJ components have been linked to an increased risk and severity of atopic dermatitis [35]. Such a predisposition might result in the decreased expression of claudin-1 and -23, causing the defect of the bioelectric barrier within the epidermis of patients with AD [17].

### 3.2. Filaggrin

As mentioned above, the stratum granulosum is also distinguished by the presence of keratohyalin granules which contain keratin, loricrin, trichohyalin, and profilaggrin [36]. Profilaggrin is a highly phosphorylated, functionally inactive precursor of filament aggregating protein (filaggrin), a crucial component of the physiological skin barrier [37,38]. Moving from the stratum granulosum to the stratum corneum, profilaggrin is gradually dephosphorylated and cleaved into 10–12 filaggrin monomers by endogenous proteases such as CAP1/Prss8 and SASPase/ASPRV1 [39,40,41]. Subsequently, filaggrin binds to keratin filaments and forms them into flattened squames that support the cytoskeleton and provide structure to the corneocytes to ensure adequate cell-to-cell adhesion [37,42]. Keratin filaments in the corneocytes are bound by corneodesmosomes formed by cytoplasmic anchoring proteins (desmoplakin, plakoglobin) and adhesion molecules (desmoglein and desmocollin) [43]. Finally, filaggrin monomers detach from keratin and are cleaved by proteases which degrade them into amino acids and their derivatives. These final products of filaggrin formation are collectively referred to as natural moisturizing factor (NMF) due to their crucial role in maintaining skin hydration [44].

### 3.3. Endogenous Proteases

The physiological differentiation of the epidermis and adequate cell turnover is largely due to the subtle balance between the activity of epidermal proteases and their inhibitors [45]. Proteases degrade intracellular connections, which is essential for the desquamation of the terminally differentiated corneocytes [38]. The increased activity of proteases or decreased expression of protease inhibitors results in the excessive degradation of the epidermal barrier [18].

According to the literature, gene polymorphisms resulting in the overexpression of certain types of kallikrein-related proteases were linked to AD [46]. Kallikreins (KLKs) coordinate the process of physical desquamation by degrading corneodesmosome components (desmoglein1, desmocollin 1, and corneodesmosin) [47]. Some of them (e.g., KLK5 and KLK7) are also involved in the modification of filaggrin and lipid processing enzymes such as ß-glucocerebrosidase and acidic sphingomyelinase [15,48]. The function of KLKs is regulated by endogenous protease inhibitors, e.g., lymphoepithelial Kazal-type-related inhibitor (LEKTI) encoded by serine protease inhibitor of the Kazal-type 5 (*SPINK5*) gene [15]. Loss-of-function mutations in the latter may lead to the uncontrolled activation of KLKs which degrade intraepithelial connections and downregulate epidermal lipids. Homozygous mutations in the *SPINK5* result in the development of Netherton syndrome, a disease characterized by the presence of atopic diathesis, severe ichthyosiform erythroderma, and hair abnormalities [49].

Importantly, the activity of endogenous proteases is also regulated by physicochemical factors, such as skin pH [50]. The latter is typically higher than physiological values in AD being partly due to microbiome disorders, which causes the excessive activity of proteases [51,52].

### 3.4. Epidermal Lipids

A frequent analogy used to describe the structure of the stratum corneum is that of “brick” and “mortar” referring to corneocytes and lipid-rich extracellular matrix, respectively [53,54]. The latter is composed of ceramides, free fatty acids, cholesterol, and triacylglycerol species. Along with corneocytes, epidermal lipids form a barrier that prevents TEWL and the penetration of irritants and allergens [54]. AD skin exhibits an altered expression of COUP-TF-interacting protein 1 and 2 (CTIP1 and CTIP2), the transcription factors regulating the activity of lipid forming enzymes [55,56]. A study conducted in CTIP1-deficient mice showed several abnormalities, e.g., significant downregulation of ketodihydrosphingosine reductase (Kdsr), UDP-glucose ceramide glucosyltransferase (Ugcg), N-acylsphingosine amidohydrolase 1 (Asah1), and ceramide synthase 6 (Cers6) [55].

AD was shown to be particularly associated with ceramide abnormalities. A report by Berdyshev et al. revealed that the overall paucity of ultra-long-chain ceramides (i.e., more than 26 carbons in length) and a relative increase in short-chain ceramides was associated with the upregulation of Th2 cytokines and epidermal barrier impairment [57]. 

Damage to the epidermal barrier triggers the compensatory production of free fatty acids (FFAs) [58]. Physiologically, FFAs show cytotoxic activity and antimicrobial properties [59]. However, as regards AD, FFAs are characterized by shortened fatty acyl chain length and an increased level of unsaturated fatty acids, which compromises their function as factors maintaining epidermal barrier integrity and the homeostasis of the cutaneous microbiota [60].

## 4. Immune Dysregulation in Atopic Dermatitis

AD is characterized by defects in both innate and acquired immune responses. A detailed description of immune abnormalities in AD exceeds the scope of this review. However, due to a close relationship between the epidermal barrier function, immune dysregulation, and microbiome dysbiosis, we present the essential concepts below.

The innate immune system is responsible for the rapid identification and neutralization of pathogenic microbes and for the recognition of host tissue damage to trigger its repair [61]. These processes are mediated by pattern recognition receptors (PRRs) that are stimulated by danger-associated molecular patterns (DAMPs) and pathogen-associated molecular patterns (PAMPs) [62]. Once activated, PRRs may activate a wide range of immunocompetent cells. AD is associated with abnormalities in the signaling via the following PRRs: (1) Toll-like receptors (TLR) [63]; (2) nucleotide-binding oligomerization domain (NOD) [64]; and some soluble PRRs [65]. These abnormalities result in the reduced reactivity of dendritic cells, polymorphonuclears, and NK lymphocytes, as well as a decreased synthesis of antimicrobial peptides (AMPs) in the skin [65]. Collectively, all these disorders hamper the control of pathogens on the skin.

With respect to acquired immune response, AD has been associated with variable dysregulation of Th lymphocytes dependent on the patient’s phenotype and disease phase [66]. In general, the overexpression of Th2 cytokines (thymic stromal lymphopoietin (TSLP), IL-4, IL-13, IL-31) is a common finding in the acute phase of the disease [9]. Th22 is another axis that is typically upregulated in AD, primarily resulting in the overexpression of IL-22 [67,68]. Both Th2 and Th22 cytokines impair the terminal differentiation of keratinocytes by interfering with filaggrin, loricrin, and involucrin modification and the production of tight junction components, particularly claudins [69]. Chronic AD lesions, apart from a high expression of Th2 and Th22 cytokines, are also characterized by the activation of the Th1 axis and upregulation of markers such as IFN-γ, CXCL9, and CXCL10 [68]. Lastly, in Asians, children, and in patients with intrinsic AD, a marked expression of Th17 cytokines is seen [66].

## 5. The Skin Microbiome in Atopic Dermatitis

Despite being a dynamic and challenging habitat, the skin is home to a highly diverse array of bacteria, viruses, and fungi collectively referred to as the microbiome [22]. With the advent of next-generation sequencing, it became possible to appreciate the diversity of bacterial species that had previously been impossible to identify with classic culturing methods. The initial studies on the skin microbiome were conducted by Grice et al. who analyzed the bacterial composition in twenty locations on the human body by targeted amplicon sequencing of the 16S rRNA subunit [70]. Four out of nineteen detected bacterial phyla were the most dominant: Actinobacteria (59% of total bacterial species belonging to the *Micrococcus*, *Propionibacterium*, *Corynebacterium* genera), Firmicutes (24%, *Lactobacillus*, *Streptococcus*, *Staphylococcus*), Proteobacteria (17%, *Paracoccus*, *Haematobacter*), and Bacteroidetes (7%, *Prevotella*, *Porphyromonas*). The composition of the skin microbiome depends on numerous intrinsic (e.g., sex, age, occupation) and extrinsic (moisture, pH, sebum content, UV exposure) factors [71]. These observations were the basis for distinguishing three primary types of microniches: (1) sebaceous (mainly inhabited by *Cutibacterium* spp.), (2) moist (characterized by the domination of *Staphylococcus* spp. and *Corynebacterium* spp.), and (3) dry (harboring diverse microbiota) [22].

Initial reports on the physiological skin microbiome were shortly followed by studies based on the genome sequencing of skin samples in various dermatoses. An investigation in AD confirmed the observations from studies implementing classic culturing methods and identified *Staphylococcus aureus* as the pathogen dominating the lesional skin of AD patients [72]. A longitudinal analysis of the skin microbiome in the pediatric population with AD demonstrated that disease flares were to a certain extent preceded by the domination of the physiological skin microbiome by *S. aureus* [73], which contributed to the hypothesis on the fundamental role of microbial dysbiosis in AD. In the mentioned report, a concomitant proliferation of *S. epidermidis* and the correlation of this phenomenon with disease severity were also demonstrated. Habitually, *S. epidermidis* was only perceived as an important skin commensal orchestrating the maturation of the immune system and combating pathogens while spontaneously causing opportunistic infections [74]. Based on the results of in vitro studies showing that *S. epidermidis* inhibited the growth of *S. aureus*, its proliferation in AD lesions was attributed to the ability to thrive in *S. aureus*-dominated habitat [75,76]. However, the latest reports suggested that in certain conditions, *S. epidermidis* might contribute to the inflammatory reaction in AD [77,78]. Additionally, a recent study implementing a 3D human skin model revealed that *S. epidermidis* elicited a strong bias toward the downregulation of genes in comparison with other bacterial species [79]. With respect to the genes of the epidermal differentiation complex, despite the upregulation of *FLG* and *LOR* encoding filaggrin and loricrin, respectively, a significant downregulation was shown for *IVL*, *FLG2*, *TCHH*, *SPRR-3*, *SPRR-4*, and *S100A7* encoding involucrin, filaggrin-2, trichohyalin, small proline rich protein-3 and -4, and S100 calcium binding protein A7, respectively. The authors hypothesized that due to its high prevalence on the skin, *S. epidermidis* might have more unique interactions with the host tissue than other microbes. The discrepancies in the presented studies may suggest that the impact of *S. epidermidis* on AD is more nuanced, and probably dependent on many variables such as strain specificity [80].

Other niches have also been investigated in AD. Similarly to skin lesions, the nonlesional skin was revealed to harbor an increased load of *S. aureus* [81,82,83]. Furthermore, a report comparing the microbial composition of the nonlesional skin of AD patients and the skin of controls demonstrated the abundance of *Streptococcus* spp. and *Gemella* spp. with the simultaneous depletion of *Dermacoccus* spp. in the former group [51]. The researchers concluded that such changes might result in the excessive production of ammonia and a subsequent increase in skin pH. As discussed above, the latter is a typical feature of AD causing disturbances in the functioning of the skin barrier.

## 6. Biofilms

Although the microbes multiply most efficiently in the planktonic form, in most cases they form multi-species communities producing extracellular polymeric substance (EPS), which enables the attachment to biotic or abiotic surfaces [84,85]. This form of bacterial life is referred to as biofilm and renders bacteria more resistant to antibiotics, unfavorable environmental conditions, and recognition by the host immune system [84,86,87]. These functions are largely related to the properties of EPS, a complex structure composed of various extracellular polysaccharides, DNA, and proteins, penetrated by channels ensuring the supply of water, air, and nutrients [85,87].

Importantly, the cycle of biofilm formation is strictly associated with the production of virulence factors by the bacteria. These phenomena are regulated by the so-called quorum sensing systems which control the biofilm formation cycle depending on cell density within the bacterial community, environmental factors, the accessibility of nutrients, and other variables [88].

**Figure 2:**
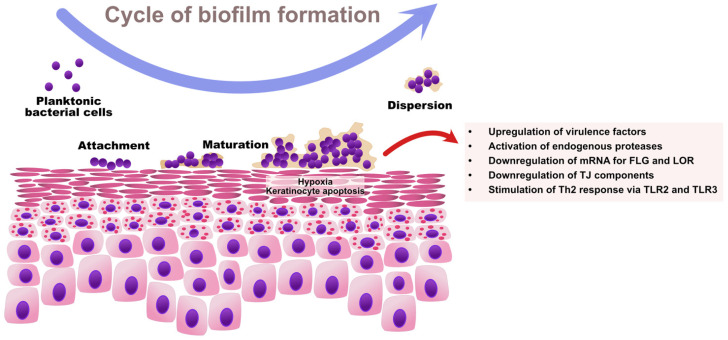
The cycle of *S. aureus* biofilm formation and its impact on skin barrier function in AD. The increased expression of adhesion molecules, epidermal lipid aberrations, and paucity of AMPs predispose AD patients to *S. aureus* colonization. Initially, planktonic *S. aureus* cells adhere to the skin surface via microbial surface components recognizing adhesive matrix molecules (MSCRAMMs) and start producing extracellular polymeric substances (EPS), which results in the establishment of a bacterial community associated in the biofilm. The growing biofilm causes hypoxia and the apoptosis of keratinocytes. Once the bacterial quorum is achieved, the Agr system activates the upregulation of virulence factors including proteases and toxins, and the downregulation of colonization and adhesion molecules with the dispersal of biofilm to new locations. Collectively, *S. aureus* biofilm contributes to the destruction of the epidermal barrier by downregulating EDC proteins and TJ components, and by stimulating Th2-skewing as a result of aberrant innate immune response. FLG—filaggrin; LOR—loricrin.

As regards *Staphylococcus* spp., the ability to sense bacterial quorum is controlled by the accessory gene regulator (Agr) [89]. An Agr product, the autoinducing peptide (AIP), is a molecule acting as an extracellular quorum signal [90]. Once a sufficiently high density of cells is achieved within a bacterial community, Agr triggers the expression of toxins and degradative exoenzymes and simultaneously causes the downregulation of colonization factors. Therefore, high Agr activity contributes to: (1) the upregulation of staphylococcal virulence factors and (2) the degradation of biofilm with the dispersal of microcolonies to new niches [91]. High Agr activity results in acute staphylococcal diseases (e.g., pneumonia), whereas low Agr activity is a characteristic feature of chronic infections mediated by biofilm (e.g., infections of indwelling medical devices) [92,93]. The cycle of biofilm formation by *S. aureus* and its impact on skin barrier in AD is presented in Figure 2.

The highly unstable environment of the skin subjected to frequent temperature and humidity changes, variable UV exposure, and bactericidal substances (e.g., in personal hygiene products) likely results in the functioning of most cutaneous microbes within biofilms. In AD, this phenomenon may also be partly responsible for the persistent microbial dysbiosis of the skin [94]. However, during the flares, *S. aureus* probably tends to degrade the biofilm and upregulate the virulence factors which triggers an even more pronounced epithelial barrier degradation and immune dysregulation.

## 7. The Influence of Barrier Disruption on Skin Microbiome Dysbiosis

Before addressing the influence of microbiota on skin barrier function, it is essential to describe the molecular mechanisms predisposing AD patients to colonization by pathogenic bacteria.

The skin barrier in AD is characterized by the abnormal expression of various surface molecules which may be used by pathogens to attach to the skin surface. For example, fibrinogen, fibronectin, and collagen may be bound by microbial surface components recognizing adhesive matrix molecules (MSCRAMMs), specialized structures developed by *S. aureus* [95]. Interestingly, it was reported that the efficient binding of fibronectin and fibrinogen by *S. aureus* was possible on AD skin and not on healthy skin or in other inflammatory dermatoses [96]. Binding via MSCRAMMs is followed by the secretion of EPS and biofilm production with the resultant establishment of a stable bacterial community on the skin surface.

Although various studies included contradictory results, it seems that microbial dysbiosis might also be associated with filaggrin deficiency. A trial by Clausen et al. revealed that *S. aureus* colonization of the lesional skin and the nose correlated with the presence of filaggrin loss-of-function mutations [97]. Furthermore, Cai et al. demonstrated that filaggrin mutations were a risk factor for skin infections in children with AD [98]. Nakatsuji et al. showed that filaggrin-deficient mice were prone to the increased penetration of the epidermis by *S. aureus* [99]. Conversely, Simpson et al. showed that despite the fact that *S. aureus* carriage correlated with the parameters reflecting epidermal barrier damage, there was no association between this phenomenon and filaggrin mutations [100].

Filaggrin deficiency is associated with the paucity of NMF. One study demonstrated that *Staphylococcus aureus* implemented clumping factor B to bind to corneocytes and that the strength of bacterium-corneocyte adhesion correlated with NMF levels [101]. The concept was further investigated by Towell et al., who showed that a low concentration of NMF resulted in the aberrant expression of corneodesmosin, a target structure bound by fibronectin-binding protein B (FnBPB) and clumping factor B (ClfB) [102]. The authors demonstrated that blocking the N-terminal region of corneodesmosin inhibited the adhesion of *S. aureus.* Furthermore, FnBPB- and ClfB-deficient *S. aureus* mutants were incapable of binding to human keratinocytes.

As mentioned above, alterations in the stratum corneum lipids are another factor predisposing to microbiome disorders in AD. A recent study by Igawa et al. showed that sphingosine-deficient mice were characterized by the increased penetration of *S. aureus* through the skin [103]. Similar observations were made in an experiment by Lipsky et al., who concluded that the stratum corneum was more easily penetrated by *S. aureus* when the lipid levels were depleted to simulate AD skin [104]. Baurecht et al. showed that apart from filaggrin deficiency, *S. aureus* abundance correlated with ceramide α-Hydroxy fatty acid/sphingosine base activity [105], while Cleary et al. demonstrated that ceramide distribution might structurally inhibit the formation of bacterial biofilms, and proposed that the characteristic pattern of their depletion typical for AD probably served as a conduit for *S. aureus* growth [106]. Lastly, a decrease in monounsaturated fatty acid species (MUFAs) seems to be an additional factor predisposing to dysbacteriosis in AD [107].

## 8. The Influence of Skin Microbiome Dysbiosis on the Skin Barrier

Microbial dysbiosis impairs skin barrier function through diverse mechanisms. Recent studies on the role of the microbiome in skin physiology revealed its influence on the expression of numerous factors implemented in skin immunity and barrier function. For example, Meisel et al. showed that immune response genes encoding TLRs, antimicrobial peptides, the complement cascade, and IL-1 family cytokine signaling were modulated by the microbiota [108]. Furthermore, the skin microbiome was revealed to influence keratinocyte differentiation and development by modifying the expression of genes in the epidermal differentiation complex (EDC).

The influence of the abnormal microbial composition of the skin is discussed with respect to the previously described elements of the skin barrier.

### 8.1. Effect on Tight Junctions

Tight junctions are targeted by microorganisms that penetrate the stratum corneum. The degradation of their components and the resultant increased skin permeability contribute to AD exacerbations.

Research in impetigo resulted in the publication of one of the first reports describing the influence of *S. aureus* on tight junction function. The authors concluded that ZO-1 and Ocln were downregulated in the infected zones and upregulated in the neighboring colonized areas of the skin [109]. More recent studies demonstrated that challenging normal human epidermal keratinocytes (NHEKs) with live *S. aureus* affected the TJ function in a biphasic way [110]. At early time points *S. aureus* was shown to strengthen the TJ function which was supported by the increased transepithelial resistance and reduced FITC–dextran permeability. The authors attributed this process to the relocation of Ocln and Cldn-4 to the cell-cell surface. However, long-term exposure caused a decrease in TJ function, probably due to the downregulation of Cldn-1 and Cldn-4. Similar albeit less pronounced effects on tight junction function were demonstrated for *S. epidermidis.*

Some studies pointed out selected staphylococcal virulence factors as the culprits of decreased TJ function. Wang et al. demonstrated that SspA/V8 protease was the primary secreted factor of *S. aureus* correlating with skin barrier permeability and tight junction damage [111]. The effect of SspA/V8 was limited by IL-1β-induced production of human β-defensin 2. However, as mentioned in the introduction, Th2 skewing typical of AD likely results in the downregulation of human β-defensin 2 and insufficient protection of TJs from staphylococcal proteases.

TJ function is largely regulated by the innate immune system. In line with these observations, Kuo et al. showed that TLR2 enhanced the production of TJ proteins, such as claudin-1, claudin-23, occludin, and ZO-1 upon the exposure to *S. aureus*-derived peptidoglycan and synthetic TLR2 agonists [112]. However, patients with AD often exhibit PRR polymorphisms that impaired those processes. Furthermore, some staphylococcal superantigens, such as SEB, were shown to diminish Ocln and ZO-1 in a model of chronic sinusitis [113] which is likely translatable to AD.

### 8.2. The Influence on Filaggrin and Epidermal Differentiation

It was shown that the microbiota might influence the differentiation and maturation of keratinocytes. According to the literature, the submission of 3D skin tissue cultures to mixed microbiota treatment resulted in the upregulation of filaggrin and epidermal cell proliferation [79]. Such an effect was not observed after skin exposure to single microorganisms. As the microbiota of AD patients is dramatically dominated by *Staphylococcus* spp., it is very likely that filaggrin deficiency in AD may be aggravated by reduced microbial diversity.

Furthermore, various studies showed that filaggrin and epidermal differentiation were impaired by Th2-cytokines, such as IL-4, IL-13, IL-31, and IL-33. At the molecular level, the downregulation of EDC components such as FLG and LOR by IL-4 and IL-13 is mediated by Janus kinases (JAK) 1 and 2, and tyrosine kinase 2 (TYK2) which induce the phosphorylation of STAT6 and STAT3 resulting in the downregulation of EDC molecules. The accessory mechanism of filaggrin downregulation involves the IL-13/periostin pathway inducing IL-24 production in keratinocytes [114]. Furthermore, IL-31-treated HaCaT cells showed differentiation defects associated with the underexpression of filaggrin, reduced epidermal thickness, and poor development of the stratum granulosum, while IL-33 was shown to downregulate filaggrin and TJ components such as claudin-1 through STAT3 and ERK phosphorylation in human keratinocytes [115,116,117,118]. Therefore, as microbiome disorders in AD are capable of triggering Th2 skewing, they also indirectly exert a negative effect on the skin barrier. Th2 polarization caused by AD-specific pathogens was proven by Nakatsuji et al., who demonstrated that *S. aureus* was capable of inducing the upregulation of IL-4, IL-13, IL-22, and thymic stromal lymphopoietin after skin penetration [99]. Moreover, Kim et al. showed that mice exposed to *S. aureus* and recombinant staphylococcal enterotoxin A showed the increased levels of proinflammatory cytokines, such as IL-4, IL-13, INF-γ, IL-17, and IL-18 [119]. Additionally, Kindi et al. discovered that *S. aureus* second immunoglobulin-binding protein (Sbi) was a type-2 promoting virulence factor that aggravated AD by triggering IL-33 expression and the downregulation of corneodesmosin [120]. Similar observations were also made in a study concerning allergic nasal mucosa, wherein *S. aureus* was shown to stimulate IL-33 and TSLP [121]. Furthermore, Brauweiler et al. showed that *S. aureus* lipoteichoic acid worked in combination with IL-4 and IL-13 through p63 to aggravate the suppression of early keratinocyte differentiation [122]. The same group revealed that the lipoteichoic acid of *S. aureus* aggravated epidermal barrier damage by reducing the expression of filaggrin and loricrin via an IL-1 mediated pathway [123]. Conversely, another study showed that the exposure to staphylococcal enterotoxin B was associated with a decreased expression of filaggrin degradation products [124]. Late keratinocyte differentiation resulting from the exposure to *S. aureus* antigens was also demonstrated in one report, in which the authors identified IL-6 as the offending molecular mediator [125].

### 8.3. Skin Barrier Disruption Mediated by Proteases

Pathogens residing on the skin may aggravate epidermal barrier defects by the secreted proteases and by modifying the activity of the endogenous proteases of the host.

According to Williams et al., *S. aureus* was shown to stimulate the activity of selected kallikreins (KLK6, 13, and 14). It coincided with the degradation of desmoglein-1 and filaggrin and an aberrant barrier function [126]. Another study revealed an increased proteolytic activity within the epidermis following the exposure to phenol-soluble modulin α derived from *S. aureus* [127].

With respect to the secreted proteases of *S. aureus* and *S. epidermidis*, apart from inducing epithelial barrier damage, they were also shown to degrade galectin-3, which orchestrates antimicrobial response in the skin, suggesting a new mechanism for staphylococcal skin colonization and immune evasion in AD [128]. *S. epidermidis* was also shown to secrete EcpA cysteine protease that has the ability to degrade desmoglein-1 and LL-37 (an antimicrobial peptide) in vitro with the resultant disruption of skin barrier function [129]. The process was dependent on quorum sensing, implying that it might be excessively activated because of the increased *S. epidermidis* density on the lesional skin of AD patients. Additionally, the observations of the murine model of *S. aureus* skin infections revealed that proteases were crucial for producing more severe lesions in the fatty acid kinase A (FakA)-deficient strain of *S. aureus*, underlying their role in orchestrating the cutaneous colonization and infection by this pathogen [130]. Lastly, staphylococcal exfoliative toxins and α-toxin were shown to degrade desmoglein-1 and E-cadherin, respectively [131].

## 9. The Influence of Skin Microbiome Dysbiosis on Itch

As discussed in the introduction, the itch–scratch cycle plays a detrimental role in aggravating epidermal barrier defect, immune dysregulation, and microbial dysbiosis in AD. However, the latest literature reports suggested that the dysbiotic skin microbiome itself was an important factor stimulating itch [132].

As regards AD, damaged keratinocytes and skin microbes upregulate pro-inflammatory cytokines, proteases, and neuropeptides that stimulate peripheral nerve fibers, thus causing the sensation of itch [132]. Seemingly, in the case of AD this process is largely triggered by *S. aureus*. Firstly, *S. aureus* antigens may upregulate IL-31, a cytokine known to mediate itch by operating directly on the sensory neurons via TRPV1 and TRPA1 [133]. Furthermore, the upregulation of serine protease activity results in cleaving protease-activated receptors (PARs) which are present on many cell types including sensory neurons [134,135]. As highlighted above, serine proteases additionally trigger Th2 inflammation, which is associated with the sensation of pruritus [136]. *S. aureus* also seems to induce histaminergic itch by causing the degranulation of mast cells in a process mediated by δ-toxin [137]. Lastly, it was shown that N-formylated peptides and α-hemolysin from *S. aureus* directly stimulated the sensory neurons and elicited the sensation of pain [138], which could imply their role in generating itch in a similar manner.

The sensation of pruritus is an important factor causing stress in AD [139]. In this context, it is also important to emphasize that the release of substance P resulting from stress modulates the virulence of Gram-positive bacteria, such as *S. aureus* [140,141].

## 10. The Effect of Bacterial Biofilms on the Skin Barrier in Atopic Dermatitis

As outlined in the introduction, biofilms constitute the most prevalent form of bacterial skin colonization. The epidermis of AD patients is a relatively beneficial habitat for the rapid development of biofilms, especially those of *Staphylococcus* spp. In the case of *S. aureus,* the endogenous propensity to form biofilm depends on the bacterial genotype. *S. aureus* strains may be grouped into clonal complexes (CCs) based on multilocus sequence typing of staphylococcal housekeeping genes or Staphylococcus protein A (spa) typing [142]. The dominant CCs colonizing patients with AD and the healthy population tend to differ, which translates into the propensity to form biofilm on the skin surface [143,144].

The pathogenic role of staphylococcal biofilms for the skin barrier is diverse. Firstly, the developing biofilm forms an impermeable barrier that causes hypoxia and the subsequent apoptosis of keratinocytes [145,146]. An in vitro study by Tankersley et al. revealed the cytotoxic effect after only 3 h of keratinocyte exposure to staphylococcal biofilm and was not present upon incubation with a sessile form of *S. aureus* [147]. Furthermore, in vivo assays showed that biofilms developing in AD skin lesions infiltrated the sweat ducts, which aggravated pruritus with the subsequent destruction of the skin barrier due to scratching [148,149]. Longstanding biofilm infections caused by *S. aureus* may also prompt the self-destruction of host tissues by the activated immune system, which perpetuates the biofilm-related disease and possibly contributes to the defect of the skin barrier [150].

A study by Gonzalez et al. revealed that the presence of *S. aureus* showing higher relative biofilm propensity compared with *S. epidermidis* was associated with increased TEWL on both the lesional and nonlesional skin of pediatric AD patients, pointing to epidermal barrier impairment in this cohort [151]. Conversely, Sonesson et al. revealed that proteases secreted late in the process of biofilm development might inactivate antimicrobial peptides, such as LL-37, and hypothesized that they might also contribute to activating kallikreins and cleaving structural proteins important for maintaining skin barrier [152]. Furthermore, according to the literature, bacterial extracts of biofilm-producing *S. aureus* reduced the expression of mRNA for loricrin and filaggrin by 70–90%, and upregulated mRNA for filaggrin-degrading enzymes caspase-14 and calpain-1 in NHKs, suggesting abnormalities in the epidermal differentiation of the upper stratum corneum [125]. Biofilms may also impair the restoration of tight junctions. Mixed bacterial biofilms were associated with the downregulation of ZO-1 and ZO-2 with a resultant increase in TEWL in a chronic wound model [25] (Figure 2).

Biofilms are additionally known to cause significant aberrations in the immune response. As outlined above (see the section about filaggrin), immune dysregulation typical of AD subsequently causes the disruption of the epidermal barrier. Biofilm-dependent keratinocyte apoptosis and *S. aureus* antigens, such as lipoteichoic acid, result in the release of numerous DAMPs which trigger innate immunity via TLRs. TLRs are then responsible for eliciting Th2-immune response with the subsequent downregulation of EDC components. In a murine model, triggering TLR2 was shown to induce chronic AD via IL-4-mediated IL-10 suppression [63]. Conversely, TLR3 activation was associated with the upregulation of TSLP, a potent factor responsible for immune polarization towards Th2 response [153].

Indirect insights into the role of biofilms for the epidermal permeability barrier come from the research on disorders affecting the mucous membranes. For example, biofilm exoproteins from *S. aureus* strains isolated from chronic rhinosinusitis patients were analyzed for their influence on primary human nasal epithelial cells grown at the air–liquid interface [154]. The study revealed the dose- and time-dependent cytotoxicity of epithelial cells, increased permeability, and the reduction of transepithelial resistance after exposure to the biofilm culture of *S. aureus* in comparison with planktonic cells. Furthermore, the disruption of tight junctions was demonstrated on electron microscopy and a decrease in zonula occludens-1 and claudin-1 was shown with immunofluorescence imaging.

## 11. Therapeutic Methods with the Potential of Restoring the Normal Skin Microbiota

The critical role of microbiome dysbiosis in AD prompts the search for treatment methods that could restore the physiological composition of the cutaneous microflora and thus alleviate the disease symptoms. It must be emphasized that antibiotics are not routinely recommended in AD because of the insufficient specificity and the possibility of generating resistant pathogens [155,156].

According to current AD guidelines, the treatment with sodium hypochlorite is recommended due to its beneficial effect in alleviating itch and disease severity with varying impact on skin microbiome in different trials [1,157,158]. A recent study by Eriksson et al. showed that sodium hypochlorite used at high concentrations (i.e., 0.02% vs the recommended 0.005%) exerted a significant anti-staphylococcal and anti-biofilm effect [159]. Therefore, it seems that high-concentration sodium hypochlorite baths could be successful in preventing microbial dysbiosis in AD, provided the safety of such treatment was established.

Various novel microbiome-oriented treatments are being elaborated on in AD. One of the concepts involves topical treatment with probiotic strains hampering the growth of *S. aureus* and supporting the physiological microbiota. For example, Nakatsuji et al. demonstrated that the antimicrobials produced by *S. epidermidis* and *S. hominis* showed selective bactericidal activity towards *S. aureus* and potentiated the antibacterial effect of LL-37, a human AMP [160]. Furthermore, Myles et al. showed that the topical application of Gram-negative *Roseomonas mucosa* bacterium resulted in a significant decrease in AD severity, itch, and a steroid-sparing effect in adults and children with AD without significant side effects [161].

*S. aureus*-specific phages and phage lysins constitute another group of medicines with a potential for use in AD. A recent study showed that a treatment with *S. epidermidis* and SaGU1, a *S. aureus* phage, showed a sustained inhibition of *S. aureus* growth [162]. The ongoing research on Staphefekt, an engineered bacteriophage endolysin showing a specific bactericidal activity towards *S. aureus*, might confirm the clinical efficacy of this approach in AD treatment [163].

Additionally, it is possible that synthetic AMPs prove successful in combating *S. aureus* in AD. Dawgul et al. showed a significant anti-staphylococcal and anti-biofilm effect of two amphibian AMPs (citropin 1.1 and temporin A) [164]. Furthermore, a phase II trial of omiganan, an indolicidin analog, revealed its potential for improving microbial dysbiosis on AD skin and a small, but significant effect on clinical scores encouraging further research on the implementation of AMPs [165].

Lastly, it was shown that systemic treatment with dupilumab reduced the proportion of *S. aureus* on both the lesional and nonlesional skin and caused the increase in the population of coagulase-negative staphylococci, such as *S. epidermidis*, *S. hominis*, and *S. saprophyticus* on the nonlesional skin [166]. A study by Lossius et al. revealed that bacterial diversity was also increased on the lesional skin of patients treated with UVB therapy [167]. Such observations reflect the close relationship between the inflammatory cascade and the microbial dysbiosis, underlying the need to diversify the therapeutic process in AD.

## 12. Conclusions

The epidermal barrier defect and microbiome disorders are closely related and may contribute to the immune dysregulation in AD with the resultant aggravation of the disease course. The latest literature data revealed a nuanced influence of the skin microbiota on various components of the skin barrier such as tight junctions, proteins belonging to the epidermal differentiation complex, and endogenous proteases either directly, or by aggravating the immune dysregulation typical of AD. Furthermore, there is increasing evidence demonstrating that such pathogenic events are largely caused by bacterial biofilms. Further studies are needed to characterize the spectrum of microbiome–host interactions with respect to the skin barrier and to lay the foundation for elaborating on novel treatment methods in atopic dermatitis.
